# Vibratome Sectioning and Clearing for Easing Studies of Cassava Embryo Formation

**DOI:** 10.3389/fpls.2020.01180

**Published:** 2020-08-04

**Authors:** Zaida Lentini, Eddie Tabares, María E. Buitrago

**Affiliations:** CINEB (Center of Specialized Natural and Biotechnological Ingredients), School of Natural Sciences, Universidad Icesi, Cali, Colombia

**Keywords:** histological studies, embryo formation, gynogenesis, doubled haploids, breeding broad crosses

## Abstract

This work describes the application of clearing on vibratome sections to study the embryo formation in cassava. This procedure provides high-resolution images and reduces significantly the number of sections that need to be analyzed per ovule. This methodology was instrumental for the development of the protocol for embryo rescue in cassava. It has been also applied to monitor the embryo formation response when optimizing seed setting from regular and broad crosses for cassava breeding. Broad crosses between cassava and castor bean (incompatible-euphorbiaceae species) were made aiming to induce doubled haploids through the elimination of the incompatible-male parent genome as done in cereals. Castor bean is widely available and provides continues supply of pollen. Our results suggest that this methodology is easy and effective to assess the response of hundreds of cassava ovules pollinated with castor bean pollen, allowing the identification of multicellular structures in the embryo sac without apparent formation of endosperm. The protocol is also useful when developing and optimizing a methodology to induce doubled haploids in cassava *via* gynogenesis or from ovules pollinated with irradiated cassava pollen.

## Introduction 

Reproductive biology is the basis for genetic improvement and a comprehensive understanding of it is essential for plant breeding, whether by conventional or biotechnological methods. Fundamental studies on plant reproductive biology include anatomical analyses by microscopy using diverse histological methods (Lentini et al., 2020a; Lentini et al., 2020b). Cassava (*Manihot esculenta* Crantz) is a major staple food in tropical regions and has diverse uses as basic material for processed food, animal feed, and industrial products. However, limited fundamental information is available for understanding basic biological processes key for the development and application of breeding tools (Ceballos et al., 2020).

The plant ovule bears the female gametophyte (*i.e.* embryo sac—ES). Knowledge on the ovule pre- and post-fertilization differentiation is a key for understanding the reproductive biology of a species. An array of methods has been used to study plant ovule and embryo formation in plants. Sections or whole-mount ovules are commonly used for microscopy analysis of ovule morphology and embryo formation (Tofanelli et al., 2019), as well as for the study of gene expression (Medzihradszky et al., 2014; Bleckmann and Dresselhaus, 2016) or protein localization and distribution (Pillot et al., 2010; León-Martínez et al., 2019). The standard histology analysis of embryo development comprises tissue preparation (including fixing and embedding samples), sectioning of thin slices with a microtome or ultra-microtome, and staining for examination by light microscopy. However, this cumbersome preparation limits the quantity of high-quality sections that can be processed. Screening a large number of samples is usually required for breeding purposes. Therefore, there is interest in methodologies that facilitate and accelerate processing of numerous samples in short period of time while maintaining the fidelity of the structures being analyzed and providing images with adequate quality for their correct interpretation.

Herr (1971) introduced the use of clearing of whole plant ovule for microscopy analysis, proving an alternative for the traditional and arduous embedded microtome sectioning technique. Young et al. (1979) did a comparative analysis to detect apomixis in grasses using two techniques. Whole-mounted pistils were cleared and analyzed by conventional microscopy, and afterwards, the same pistils were embedded in and sectioned into thick slices for a second microscopy analysis. Results indicated that both methodologies provided high quality images resulting in similar interpretations, but the clearing technique was easier and reduced the time for processing the samples by 90%. Since then, clearing protocols have been used to analyze ovule, ES, and embryo development. Various basic studies aimed at understanding the reproductive biology in several species (Herr, 1982; Herr, 1992; Musiał et al., 2015; Wilkinson and Tucker, 2017; Barke et al., 2018; Hoshino et al., 2018; Colono et al., 2019; Tofanelli et al., 2019); as well as for applied studies for plant breeding (Ogburia and Adachi, 1994; Ogburia and Adachi, 1995; Ogburia and Adachi, 1996; Ponitka and S´lusarkiewicz-Jarzina, 2004; Janowicz and Wojciechowski, 2010; Hoshino et al., 2018; Kwiatkowska et al., 2019). Clearing techniques have also been used to study morphology and gene expression for understanding developmental and other biological processes (Kurihara et al., 2015; Musielak et al., 2016).

Ogburia and Adachi (1994) were the first to use clearing to study the ES in cassava. They used whole-mount cleared-pistils to detect malformation of ESs in cassava clones. They concluded that the methyl salicylate treatment yielded more accurate interpretations of morphological features of cassava ESs than using benzoate alone. Later, they used clearing to understand compatibility barriers in cassava as an appropriate integral component strategy towards the improvement of hybrid seed production (Ogburia and Adachi, 1995). These authors also used clearing to study the female gametophyte formation and to discern meiotic diplospory in cassava (Ogburia and Adachi, 1996). However, in these studies, they reported the used of mechanically isolated whole nucellus with intact ES from the integuments and then cleared the tissues instead of using complete cleared-ovules (Ogburia and Adachi, 1995); or they complemented the analysis with standard stained microtome sections (Ogburia and Adachi, 1996). It appears that their clearing technique of intact whole ovules did not have the resolution needed for this type of analyses. The clearing method depends on the studied plant tissue. The chemicals used for clearing may affect the consistency of the tissues, making their handling difficult for microscopy observations. Hence, the clearing methodology must be modified accordingly to the tissues due to the diversity of cell sizes, tissue chemistry, and density among different species (Kwiatkowska et al., 2019).

Cassava ovules are much larger compared to other species. Therefore, several histology sections are required in order to reconstruct a particular developmental stage. Cassava ovules are about 2.5–5.0 mm long and 1.0–2.0 mm wide, depending on the stage of development (Lentini et al., 2020a; Lentini et al., 2020b). In contrast, the average size of angiosperm ovule is about 0.5 mm with a variation from 0.15 mm to 2 mm (Endress, 2011). The larger the ovules, the larger the number of sections that needs to be analyzed to do a proper histological documentation. Therefore, in the case of cassava ovules and using 10 μm microtome sections, it is necessary to analyze hundreds of sections to reconstruct the images of single ovule internal structures including the ES.

In contrast to microtome, vibratome allows for production of thicker sections that provide high fidelity images of the 3D structures and cellular organization of the studied tissues (Prieto et al., 2007). Vibratome sectioning does not need fixing and embedding the samples, but the tissue must have the adequate firmness and size. Vibratome sectioning is versatile. It has been coupled with confocal microscopy (Aragon-Alcaide et al., 1998), immunohistochemical staining (Shim, 2011), fluorescence *in situ* hybridization (Martinez-Perez et al., 2000; Prieto et al., 2007), localization of gene expression (Athman et al., 2014; Xiao et al., 2019), custom designed 3D printed molds, and/or specialized software to generate high resolution 3D images (Atkinson and Wells, 2017). In the case of cassava, until now, vibratome sectioning has been coupled with immunohistochemical staining to circumscribe the site of infection of cassava brown streak virus and to investigate its effect in the cassava leaf morphology (Saggaf et al., 2019).

The work presented here describes a protocol that combines vibratome sectioning and clearing to facilitate studies of cassava embryo formation from the early stages through its complete development. These techniques can also be applied to monitor the frequency of embryo formation when optimizing seed setting from broad crosses in cassava aided by embryo rescue, which is highly genotype-dependent. The protocol is also useful when developing and optimizing a methodology to induce doubled haploids in cassava *via* gynogenesis, ovules pollinated with irradiated pollen or from interspecific/intergeneric crosses.

## Methods

### Materials and Equipment

FAA solution [38% formaldehyde: glacial acetic acid: 75% ethanol; 7:5:88, v/v)] (see section “*Sample Fixation and Dehydration for Sectioning with Vibratome*”)

Ethanol (70, 85, 96, and 100%) (see section “Sample Fixation and Dehydration for Sectioning with Vibratome”)

Agarose-block 7% (see section “Sample Fixation and Dehydration for Sectioning with Vibratome”)

Superglue (see section “Sample Fixation and Dehydration for Sectioning with Vibratome”)

Leica series 1,000 S vibratome (see section “Sample Fixation and Dehydration for Sectioning with Vibratome”)

Multi-well plate (see section “Sample Fixation and Dehydration for Sectioning with Vibratome”)

Absolute ethanol:methyl salicylate (1:1 and 1:3), and methyl salicylate 100% (see section “*Vibratome Sections Clearing or Staining*”)

Nikon Eclipse Ti S inverted microscope equipped with Nomarski’s differential interference contrast (DIC) optics (see section “*Microscopy and Imaging Processing*”)

High-resolution Nikon DS-Fi1 5-megapixel cooled color digital microscope camera, coupled with a NIS-Element imaging (see section “*Microscopy and Imaging Processing*”)

### Plant Material and Growth Conditions

Cassava hand-pollinated ovules containing embryos at different stages of development were used to establish the protocol for sample sectioning with vibratome in combination with tissue clearing or staining. The elite clone SM1219-9 from the cassava-breeding program at the International Center of Tropical Agriculture (CIAT) was used. Plants were grown in the field at CIAT Experimental Station in Palmira (Colombia), with optimal nutritional and phytosanitary conditions, rain-fed and irrigated when required, and with mean daily temperatures of 27°C (day) and 18°C (night). Female cyathia of healthy-looking and vigorous plants of similar morphology and developmental stage from the third or fourth flowering events were used for the experiments.

### Sample Collection

Day of pollination and anthesis day were assumed to be the same (Lentini et al., 2020b). Day after anthesis (DAA), instead of day after pollination (DAP), was used as reference to predict the developmental stages of zygotic embryo formation. Hand-pollinated cyathia were used to collect ovules with embryos at different stages of development. Female cyathia were selected, hand-pollinated, collected (at varying DAA), and transported to the laboratory as described by Lentini et al. (2020b). The isolating bags were kept on the hand-pollinated cyathia until samples were collected to prevent undesirable pollinations. Fifteen cyathia (45 ovules) per date (7, 14, 21–24 DAA) and treatment (as described below) were collected and analyzed. This sample size proved to be adequate for studies of cassava ES (Lentini et al., 2020a) and embryo development (Lentini et al., 2020b).

### Sample Fixation and Dehydration for Sectioning With Vibratome

Two different fixation and dehydration methods were tested to assess which one provided the best quality for sectioning cassava ovules with vibratome (**Table 1**).

**Figure 1:**
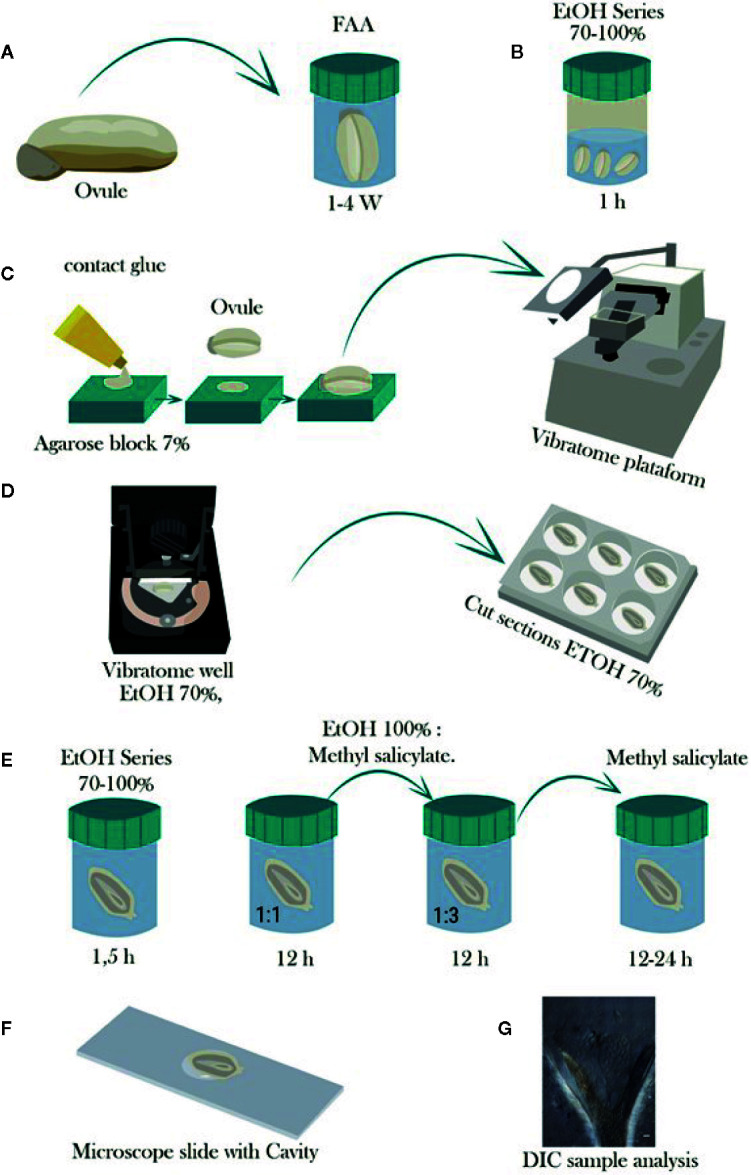
Schematic representation of the steps involved in the tissue preparation, sectioning with vibratome, and tissue clearing of cassava ovules. **(A)** Ovules are fixed in FAA for at least 1 week and stored in 70% ethanol. **(B)** Ovules are then dehydrated in a graded ethanol series from 70 to 100% and stored until use for sectioning. **(C)** Dehydrated ovules are glued onto a hand-made cavity in a 7% agarose block, and the agarose block is mounted onto the vibratome. **(D)** The tissue sectioning is performed using the vibratome at a frequency of 60 Hz and 0.4 mm. s^−1^. The sectioning is conducted under a solution of 70% ethanol to avoid curling of the slices, which are collected in multiwell plates containing 70% ethanol as well. **(E)** Afterwards, the slices are further dehydrated in a graded ethanol series up to 100% ethanol for either subsequent staining treatment or clearing in graded 100% ethanol:methyl salicylate series of 1:1 and 1:3 for 12 h each and then 100% methyl salicylate for at least 12 to 24 h. **(F)** Tissue is mounted in a microscope slide with cavity and observed in a light microscope when staining treatment is performed, or **(G)** observed with Nomarski’s differential interference contrast (DIC) optics when samples were cleared with methyl salicylate.

- Samples were fixed in FAA [38% formaldehyde (Sigma-Aldrich F8775): glacial acetic acid: (Sigma-Aldrich 537020): 75% ethanol; 7:5:88, v/v)] at least for 8 days. Then, samples were washed for a minimum of 24 h in 70% ethanol to elimite most of formaldehyde and acetic acid and stored in 70% ethanol at 4°C (Lentini et al., 2020a; Lentini et al., 2020b, **Table 1**, **Figure 1A**). After fixation, samples were dehydrated in a graded ethanol series (85, 96, and 100%—1.5 h each). After dehydration, samples were stored in 100% ethanol at 4°C until used for vibratome sectioning (Lentini et al., 2020b, **Table 1**, **Figure 1B**).
- Samples were fixed using 4% formaldehyde with infiltration (Prieto et al., 2007) or without infiltration (Wegel et al., 2005) and stored in TBS at 4°C (Prieto et al., 2007, **Table 1**). After fixation, samples were dehydrated in a graded ethanol series from 10 to 100% and stored in 100% (v/v) ethanol at 4°C until use for sectioning (Wegel et al., 2005, **Table 1**).

**Table 1:** Treatments evaluated to improve sectioning of cassava ovules with vibratome for tissue clearing or staining.

Treatment	Procedure		Notes
Fixation	In FAA for at least 8 days. Stored in 70% ethanol at 4°C^a^	In 4% formaldehyde with^b^ or without^c^ infiltration. Stored in TBS at 4°C^b^	FAA preserved well tissues with the proper consistency for sectioning slices of 60 to 150 μm from ovules of 7 DAA using vibratome
Sample dehydration	Graded ethanol series from 70 to 100%. Stored in 100% ethanol at 4°C until use for sectioning^a^	Graded ethanol series from 10 to 100%. Stored in 100% (v/v) ethanol at 4°C until use for sectioning ^c^	Starting dehydration from ethanol 70% increases time efficiency
Sample mounting or embedding	Fixed ovule onto a 7% agarose block using superglue (Aragon-Alcaide et al., 1998) and then mount the agarose block onto the vibratome stage also using superglue (this work)	Without mounting^b,c^ or embedding for sectioning in 4% low melting agarose^d^, or 5 or 7% low melting agarose (this work)	Unmounted samples were difficult to handle and to maintain in a fixed position with respect to the vibratome blade. Embedded samples usually got loose from the agarose blocks during sectioning. Fixed samples with super glue were kept steady onto the vibratome stage, increasing the control during sample sectioning
Sectioning with vibratome	Sections were cut under 70% ethanol. Then, slices were further dehydrated in a graded ethanol series up to 100% ethanol for subsequent clearing or staining treatments (this work)	Sections were cut under water^b,c^ or PBS buffer^d^	Samples were too rigid to be cut under 100% ethanol or too soft under water or PBS buffer. Cutting under 70% ethanol provides a gradual hydration during sectioning and the proper consistency, making the samples more manageable and avoiding the curling of the slices

^a^ Lentini et al. (2020a); Lentini et al. (2020b). ^b^ Prieto et al. (2007). ^c^ Wegel et al. (2005). ^d^ Shim (2011).

### Vibratome Sectioning

Preliminary experiments indicated that handling cassava ovules for vibratome sectioning could be difficult due to its large size and consistency. Ovules could easily change the position during the sectioning, and slices tended to curl. Therefore, various methodologies were evaluated to identify the procedure providing the best consistency, making the samples more manageable and avoiding the curling of the slices (**Table 1**). Before sectioning, the dehydrated ovules were either:

1. Not mounted at all before sectioning (Wegel et al., 2005; Prieto et al., 2007, **Table 1**);
2. Embedded in 4% low-melting agarose (Shim, 2011) or in 5 or 7% low-melting agarose (**Table 1**); or
3. Fixed onto a 7% agarose block using superglue (Aragon-Alcaide et al., 1998) and then the agarose block was mounted onto the vibratome stage also using superglue (**Table 1**, **Figure 1C**).

The tissue sectioning was performed using a Leica series 1,000 S vibratome at a frequency of 60 Hz and 0.4 mm. s^−1^. Longitudinal sections from 80 up to 150 µm thick were processed depending on the age of the ovule (DAA). For vibratome sectioning, the slices were cut either (**Table 1**):

- Under water (Wegel et al., 2005; Prieto et al., 2007);
- Under PBS buffer (Shim, 2011); or
- Under 70% ethanol and the slices were collected in a multi-well plate containing 70% ethanol, which avoided the curling of the slices (**Figure 1D**).

### Vibratome Section Clearing or Staining

Clearing was performed using the protocol optimized by Young et al. (1979) for grasses with some modifications as indicated below. After sectioning, the vibratome slices were dehydrated further in a graded ethanol series (85, 96, and 100%—1.5 h each **Figure 1E**). Thereafter, samples were either cleared or stained. Samples were cleared in a graded series of absolute ethanol:methyl salicylate (1:1 and 1:3, v/v for 12 h each) and then in 100% methyl salicylate for 24 h (**Figure 1E**). Sections of 50–100 µm were rinsed twice in 100% methyl salicylate, while sections of 100–150 µm were rinsed three times. Finally, the sections were left in 100% methyl salicylate until microscopy analysis.

For staining, vibratome sections were first dehydrated in a graded ethanol series (85, 96, and 100%—1.5 h each) and then stained with Safranin O–Fast Green according to Fernandez-Luqueno et al. (2008).

### Microscopy and Imaging Processing

Ovule slices sectioned with vibratome of the same DAA age were compared with slices sectioned with microtome. For microtome sectioning, ovule samples were fixed in FAA then dehydrated in a gradient of absolute ethanol:tertiary butanol (Sigma-Aldrich 471712) in seven steps of 1.5 h each, ending with pure tertiary butanol (Lentini et al., 2020a). Thereafter, sections were embedded in paraffin blocks according to Ruzin (1999). Longitudinal, 4 or 10 µm thick, sections (depending on the DAA age) were made with an American Optics Spencer 820 Rotary Microtome. Sections were placed on microscope slides, dried at 60°C for at least 1 h, and stained with Safranin O–Fast Green (Fernandez-Luqueno et al., 2008).

Vibratome sections were placed on a concave cavity microscope slide for observation (**Figure 1F**). Cleared sections were mounted in 100% methyl salicylate under an unsealed cover slip, whereas stained sections were mounted in 1–2 drops of xylene under sealed cover slip. Stained microtome or vibratome samples were analyzed under light and dark field illumination with a Nikon Eclipse 55i microscope. Vibratome samples cleared with methyl salicylate were analyzed using a Nikon Eclipse Ti S inverted microscope equipped with Nomarski’s differential interference contrast (DIC) optics (**Figure 1G**), with appropriate filters for optimal viewing. Photographs were taken with a high-resolution Nikon DS-Fi1 5-megapixel cooled color digital microscope camera, coupled with a NIS-Element imaging for acquisition, analysis, and visualization of the microscopy data.

### Validation of the Method: Vibratome-Clearing to Assess Response to Interspecific Pollination With Castor Bean (*Ricinus communis*)

Cassava cyathia were covered with cloth bags one day prior anthesis. At anthesis day, cyathia were pollinated manually with *Ricinus comunis* (castor bean). One day after pollination stigma were sprayed (or not) with water or with a solution of 2,4-D 50 mg/L and covered back with the cloth bags for a total of up to 4 days. Cyathia hand-pollinated with castor bean pollen were harvested 12 DAA and carpels cultured *in vitro* on MS3 medium according to Lentini et al. (2020b) (**Supplementary Table 1**). Thirty petri dishes containing 12 ovules per dish (for a total of 360 ovules) were cultured for each treatment. One month after culture, the ovules were excised from the carpels and sub-cultured on fresh MS3 medium, and grown under the same environmental conditions as described by Lentini et al. (2020b) (**Supplementary Table 1**). Histology analyses were conducted using 30 ovules per treatment chosen at random according to Lentini et al. (2020a). Ovule samples were fixed in FAA, dehydrated in a graded ethanol series from 70 to 100% then anchored onto a 7% agarose block using superglue. Finally, the agarose block was fixed with superglue onto the vibratome stage. Vibratome sections were cut under 70% ethanol, the slices were collected in a multi-well plate containing 70% ethanol, and thereafter, the sections were further dehydrated in a graded ethanol series up to 100% ethanol for subsequent clearing up to 100% with methyl salicylate. Microscopy and imaging processing were conducted as described above for the cleared vibratome sections.

## Results

### Vibratome Sectioning, Clearing and Staining of Cassava Ovules

Of the two fixation/dehydration methods evaluated, the fixation with FAA provided tissues with sufficient rigidity to facilitate their sectioning by vibratome (**Figures 1A, B** and **Table 1**). In contrast, samples fixed with 4% formaldehyde did not have a proper consistency and were torn apart during sectioning with the vibratome. Starting dehydration from ethanol 70% as described in the first method, rather than at 10% as in the second method, increases time efficiency for processing larger number of samples.

**Figure 2:**
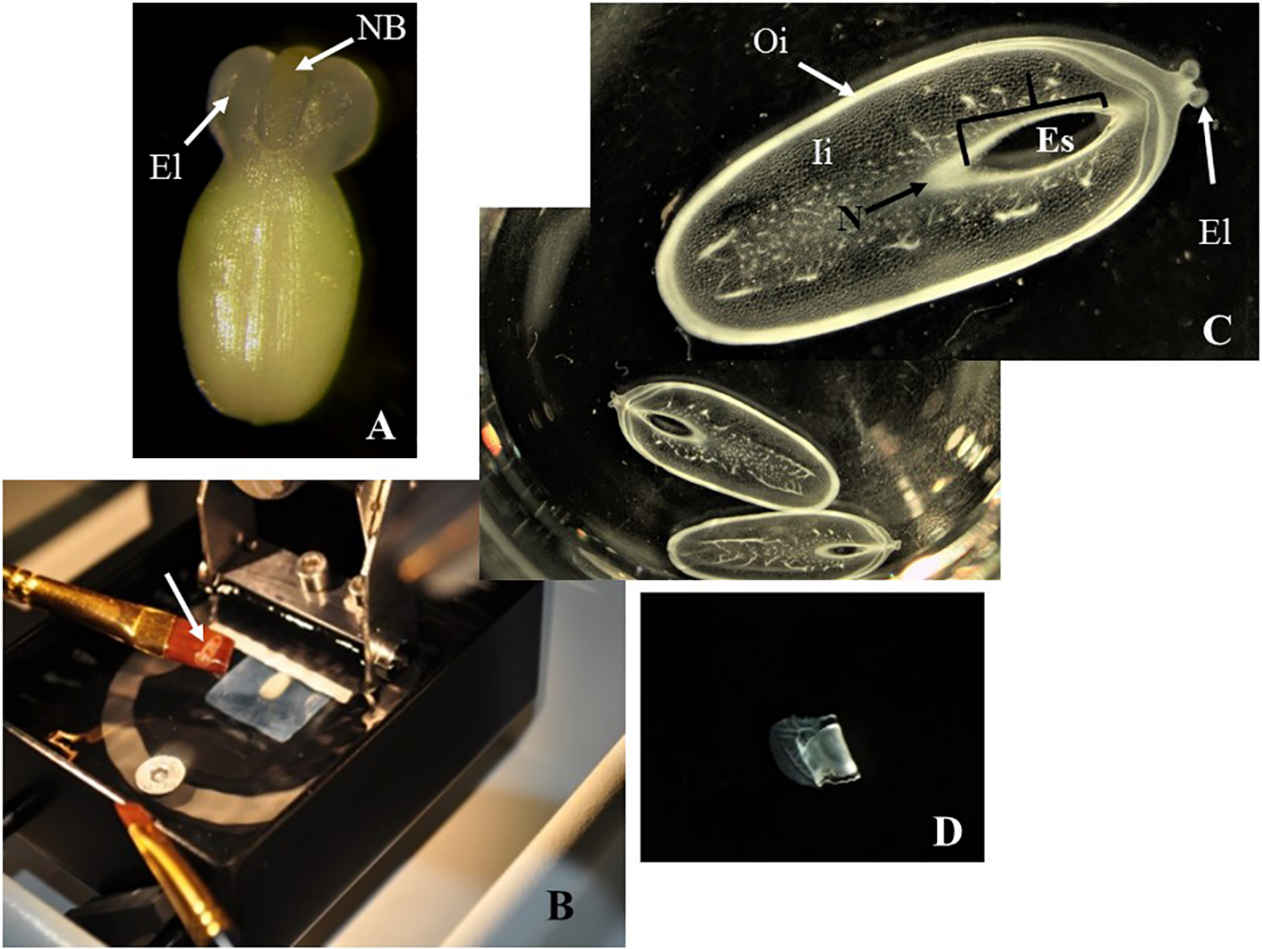
Sectioning of cassava ovules with vibratome. **(A)** A freshly dissected ovule at the day of anthesis. Large elaiosomes (El) and nucellar beak (NB) are visible. **(B)** Fixed ovule mounted onto a 7% agarose block being sectioned with vibratome. White arrow shows an ovule slice rescued on a brush. **(C)** Longitudinal sections of ovules being cut under 70% ethanol, which provides the proper consistency, making the samples more manageable and avoiding the curling of the slices. The slices were collected in multiwell plates for further dehydration and subsequent clearing or staining. Embryo sac (Es), nucellar beak (NB), nucellus (N), outer integument (Oi), and inner integument (Ii) are visible in these sections without clearing or staining. **(D)**. Longitudinal sections of ovules after being cut under 100% ethanol. These slices were rigid and curled after sectioning. **(C, D)** Longitudinal sections of 150 µm thick. Photos were taken at 10× using a light microscope.

Unmounted samples (**Figure 2A**) were difficult to handle and to maintain in a fixed position with respect to the vibratome blade. Embedded samples usually got loose from the agarose blocks during sectioning. However, fixed ovules mounted onto a 7% agarose block with super glue (**Figure 1C**) were kept steady onto the vibratome stage, improving the handling of the samples during the sectioning (**Figure 2B** and **Table 1**).

Mounted ovules onto a 7% agarose blocks became too soft under water or PBS buffer during sectioning (**Table 1**). Conversely, cutting under 70% ethanol provides a gradual dehydration of the ovule samples, making them more manageable (**Figure 1D**) and avoiding the curling of the slices (**Figure 2C**). In contrast, sections cut under 100% ethanol were too rigid and curled after sectioning (**Figure 2D**).

Vibratome sections were dehydrated further before staining or clearing (**Figure 1E**). Staining of vibratome sections was only possible in ovules containing embryos at least at the globular stage of development. Staining of vibratome samples containing earlier stages of embryo development was cumbersome, and the manipulation caused losing many samples. Staining of vibratome samples, therefore, is not practical for assessing embryo development in cassava (data not shown). On the other hand, vibratome samples cleared with methyl salicylate (**Figure 1E**) provided high quality sections for microscopy analyses and assessment of embryo development from early stages onwards (**Figure 1F**).

### Comparison of Histological Analyses of Embryo Stage of Development in Stained Microtome and Cleared Vibratome Ovule Sections

For the initial analyses we used microtome sections of 4 or 10 μm and vibratome sections than ranged from 50 to 150 μm; 10 μm-microtome sections and 130 μm-vibratome sections proved to be of high quality to identify cassava embryo development from very early stages onwards (Lentini et al., 2020b). Therefore, for this study 10 μm-microtome sections and 130 μm-vibratome sections were selected for the comparison of the two methods.

**Figure 3:**
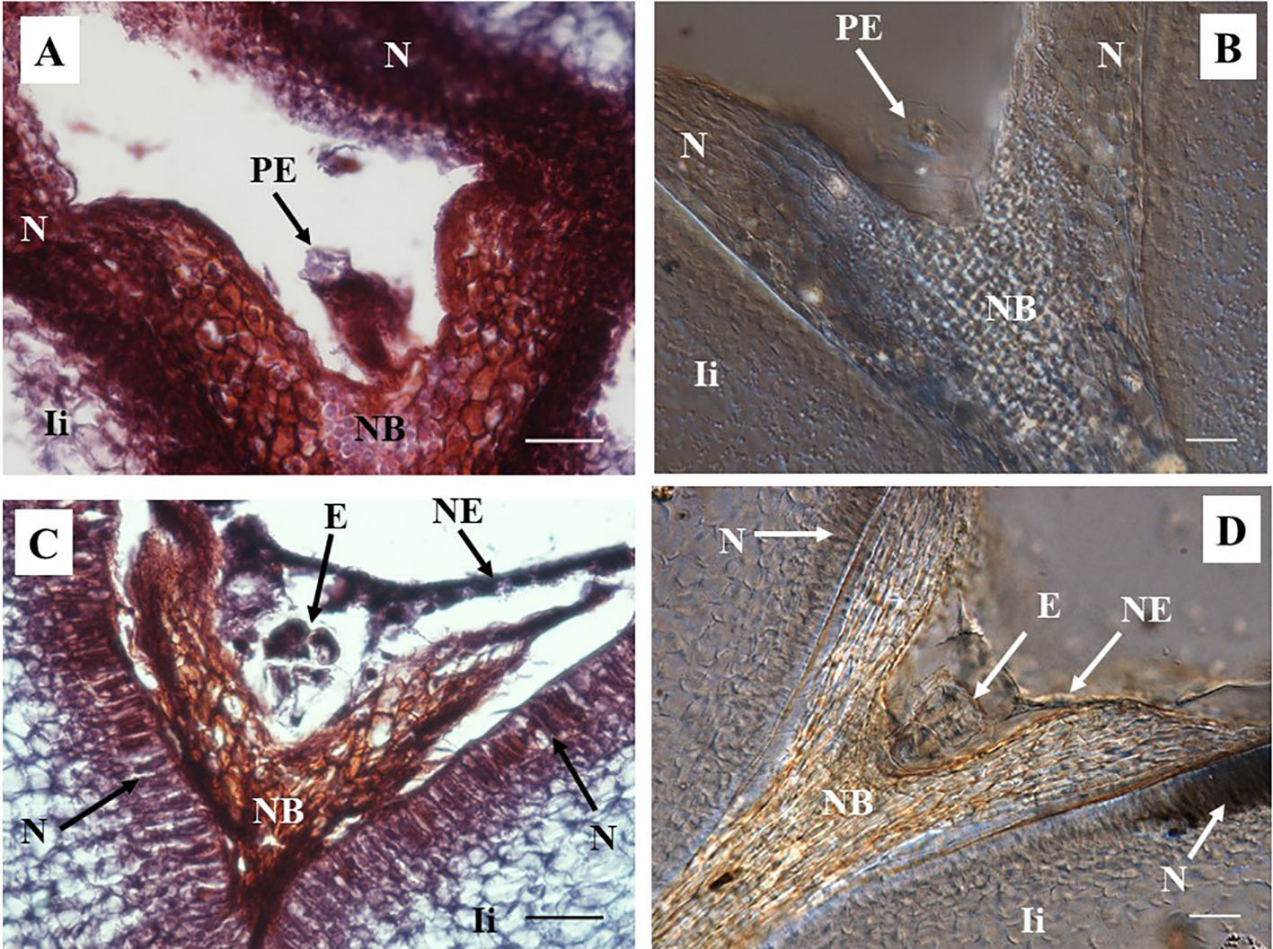
Representative images of sections containing pro-embryo and pre-globular stages of developments from hand pollinated ovules. **(A, B)** Sections of ovules collected 7 days after anthesis (DAA) show pro-embryo stage of development. **(C, D)** Sections of ovules collected 14 DAA show embryo at pre-globular stage; nucellar tissue degradation and the first signs of nucleated endosperm are also noted at this stage. **(A, C)** Longitudinal sections of 10 µm thick were processed with microtome and stained with Safranin-O and Fast Green. Photos were taken at 40× using a light microscope. **(B, D)** Sections of 130 µm were processed with vibratome, cleared with methyl salicylate, and observed with Nomarski’s differential interference contrast (DIC) optics. **(C)** Source: (Lentini et al., 2020b). Photos were taken at 40×. Bar, 20 μm. PE, pro-embryo; E, embryo; NB, nucellar beak; N, Nucellus; NE, nucleated endosperm; Ii, inner integument.

**Figure 4:**
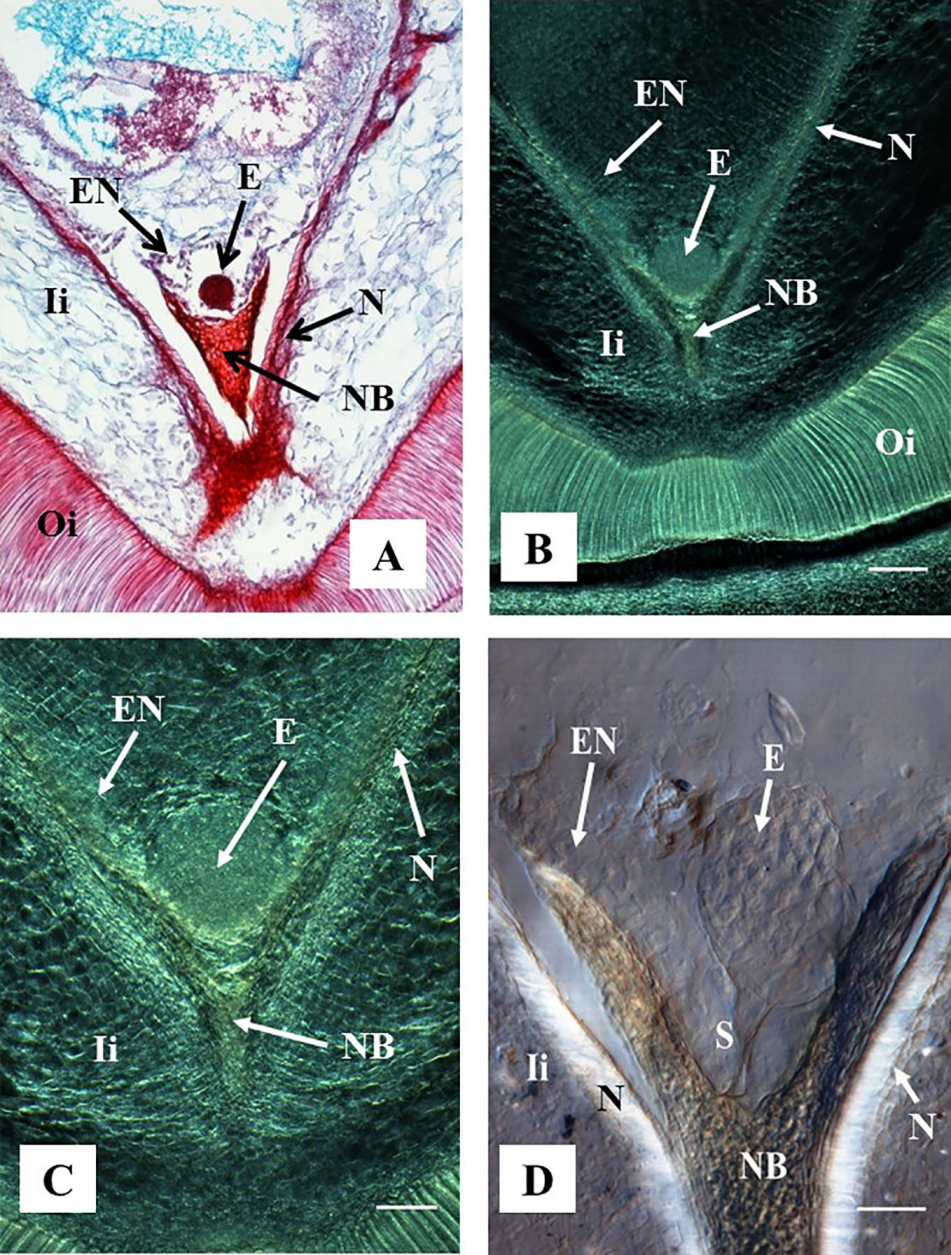
Representative images of ovule sections containing globular stage of development. Hand-pollinated ovules were collected three weeks after anthesis. **(A)** Longitudinal section of 10 µm thick were processed with microtome, stained with Safranin-O and Fast Green, and observed using a light microscope. Source: (Ramos et al., 2012). **(B–D)** Longitudinal sections of 130 µm were processed with vibratome. **(B, C)** Sections did not have further treatment and were observed using a contrast phase in a light microscope. **(D)** The section was cleared with methyl salicylate and observed with Nomarski’s differential interference contrast (DIC) optics. Source: Lentini et al., 2020b. **(A, B)** At 10×. Bar: 200 μm. **(C, D)** At 20×. Bar: **(C)** 100 μm and **(D)** 50 μm. All the sections show embryos at the globular stage of development. At this stage, the endosperm is apparent, the nucellar beak and the nucellus show significant degradation compared to earlier stages of development as shown in **Figure 3**. The outer and inner integuments are thickening, and the endosperm is fully formed. **(D)** Shows the embryo suspensor clearly visible between the embryo and the nucellar beak. E, embryo; NB, nucellar beak; N, Nucellus; EN, endosperm; Ii, inner integument; Oi, outer integument; S, suspensor.

A pro-embryo stage of development was noted in samples collected 7 DAA (**Figure 3**). The first cell divisions were clearly visible in both, stained microtome as well as cleared vibratome 7-DAA ovule sections (**Figures 3A, B**). Both methods revealed similar stages of development of other structures of the ES as well (*i.e.* the nucellar beak, the nucellus, and the inner integument, **Figures 3A, B**). However, the egg cell divisions were better discerned in the cleared vibratome sections (**Figure 3B**). Samples of 14-DAA processed with both methods allowed the identification of embryos at pre-globular stage of development (**Figures 3C, D**). The embryo cells were better distinguished in the stained microtome section (**Figure 3C**). The nucleated endosperm formation was also seen with both methods, and the nucellar tissue showed early signs of degradation (**Figures 3C, D**). The proper globular stage of development was easily identified in 21–24-DAA samples regardless of the method used (**Figures 4A–D**). At this stage, the nucellus was highly degraded, the outer and inner integuments were thick, and the endosperm was clearly identified. Better definition of the endosperm cells and the embryo suspensor was discerned in the cleared vibratome sections (**Figure 4D**).

### Analysis Using Vibratome-Clearing Sections to Assess Response of Cassava Ovules to Interspecific Pollination of Cassava with Castor Bean Pollen

**Figure 5:**
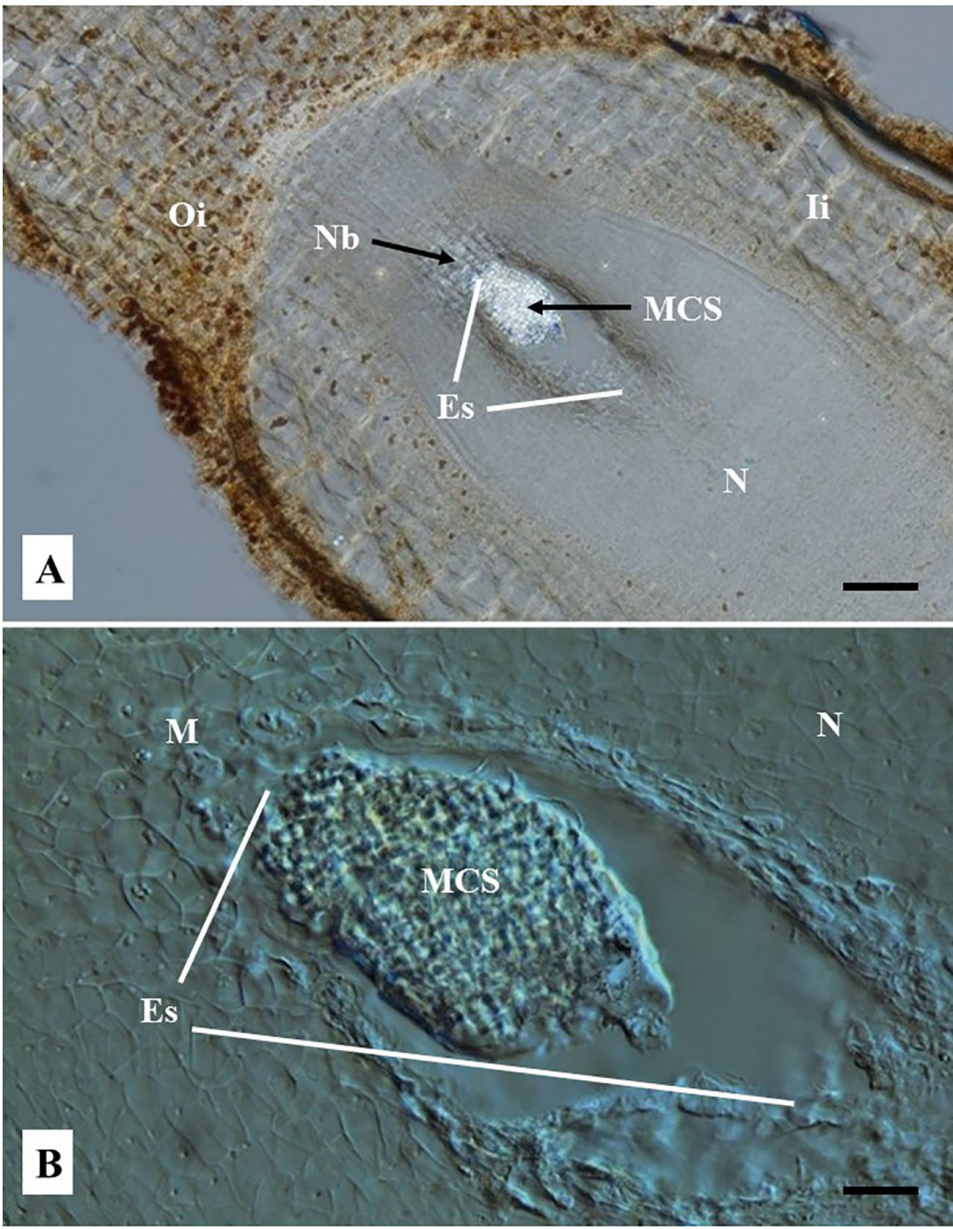
Representative images of embryo sac (ES) of cassava ovules pollinated with castor bean pollen and cultured *in vitro* 12 days after anthesis. Cyathia were hand-pollinated with castor bean (*Ricinus comunis*) one day after anthesis. Longitudinal ovule sections of 80 µm thick were obtained with a vibratome and cleared with methyl salicylate. **(A)** Sections were observed using a light microscope at 10×. (Bar = 200 μm). **(B)** Sections were observed using Nomarski’s differential interference contrast (DIC) optics at 40×. (Bar = 50 μm). **(A)** Shows a clearly distinguishable bright multicellular structure (MCS) inside the embryo sac (ES). The rest of the tissues are opaque. **(B)** Shows the MCS at the mycropylar (M) end (top end of ES in the picture). MCS appears to be composed of significantly smaller cells respect to the cells of the surrounding nucellus tissue. **(A, B)** Note the nucellus does not show any sign of degradation and the lack of endosperm inside the embryo sac, which are typically seen when zygotic embryo product of fecundation is developed. The outer and inner integuments and the nucellar beak are clearly noted. Oi, Outer integument; Ii, Inner integument; M, Micropyle end; N, nucellus; NB, nucellar beak; N, Nucellus; Es, embryo sac; MCS, multicellular structure.

Embryo sacs of ovules not sprayed at all, or sprayed with water one day after pollination with castor bean pollen, were totally empty after 4–5 months of culture. In contrast, about 5% of 1,609 cultured ovules sprayed with a solution of 2,4-D 50 mg/L showed the development of a multicellular structure inside the ovule ES (**Figure 5**). The exemplary cleared-vibratome images shown in **Figure 5** revealed a multicellular structure composed of cells that appear to be actively dividing, as indicated by the dense cytoplasm suggested by the high reflecting brightness (**Figure 5A**), their prominent nuclei, lack of vacuoles, and smaller cell sizes (**Figure 5B**). In contrast, the inner and outer integuments (**Figure 5A**) and the nucellus tissue surrounding the ES (**Figure 5B**) showed non-dividing cells with normal size nuclei, large vacuoles, and thick cell walls. Ovules with zygotic embryos actively developing in the ES are characterized by the presence of endosperm, significant degradation of the nucellus and thick-walled cells in the outer and inner integuments (**Figure 4**). In contrast, ovules pollinated with castor bean pollen appear to contain multicellular structures developed in the ES, without endosperm formation, degradation of the nucellus tissues, nor visible changes in the outer and inner integuments with respect to freshly isolated ovules (**Figure 2C**).

## Discussion

Cleared vibratome-ovule samples with methyl salicylate provided high quality sections for microscopy analyses. The information obtained, regarding the stage of embryo development from ovules of the same DAA age, was similar independently of the method used, microtome/staining or vibratome/clearing. Both methods revealed similar stages of development of other structures of the ES and the ovule as well. The optimized vibratome-sectioning/clearing methodology allows the assessment of cassava embryo development as early as the pro-embryo stage. Although, in general, the resolution of the cell images obtained with the thinner microtome sections is high, the surface texture images obtained with the vibratome sections provide volumetric contrasts and 3D-like appearance related to the different structures, unrevealed by the microtome sectioning. Visualization of the different structures was easier in the vibratome sections.

Initially, experiments were conducted aiming to use clearing alone without sectioning cassava ovules. However, the resolution of the images obtained was poor. Cassava ES is surrounded by thick outer and inner integuments (Lentini et al., 2020a; Lentini et al., 2020b), making it difficult to visualize structures developed in the ES of cleared ovules. The power of image resolution using clearing alone can be increased by merging optical sections or constructing composited images captured in a series of optical sections as demonstrated by Wilkinson and Tucker (2017). Nonetheless, our results suggest that the quality and level of resolution obtained with vibratome sectioning coupled with clearing provide high-resolution image of cassava ES, embryo development, and other ovule structures, without the need of additional image processing.

Vibratome sectioning does not require embedded samples, and it is easy to use, making it an ideal assistant tool for breeding programs. The technique permits the use of larger specimens, thus allowing the analysis of larger number of biological replicates per unit of time. Cassava ovules are large (2.5–5.0 mm long, 1.0–2.0 mm wide, Lentini et al., 2020a; Lentini et al., 2020b). Depending on the embryo stage of development, the cassava ES is located between 300 and 800 μm from the external border of the ovule outer integument. In the case of cassava ovules, when using 10 μm-microtome sections, it is necessary to analyze about 150–160 microtome-sections per ovule to reconstruct the images for the internal structures of the whole ovule and about 30–80 microtome-sections to reconstruct the images for the ES. Conversely, 12 vibratome-sections per ovule are required to reconstruct the images for its internal structure. ES information could be reconstructed analyzing images from just 2 to 6 vibratome-sections per sac. In summary, the proposed technique allows a 13 fold reduction in the number of sections required to be analyzed without loss of relevant information. Moreover, the process for the sample preparation for microtome sectioning and staining, as a whole, is considerably more time-consuming with respect to vibratome. The proposed technology ultimately results in a significant increase in the number of samples that can be analyzed per unit of time, without a reduction in the quality of the information obtained.

Vibratome sectioning, in combination with clearing, provides high quality and level of resolution of structures that, additionally, are often easier to interpret. This methodology was instrumental for the development of the protocol for embryo rescue in cassava (Lentini et al., 2020b). In cassava, ovules from the same ovary show asynchrony in development (Lentini et al., 2020a), and the success for recovering fully developed seeds from hand pollinations varies highly, depending on the genotype and environmental conditions (Yan et al., 2014). Therefore, this methodology can also be applied to monitor the embryo formation response when optimizing seed setting from regular and broad crosses for cassava breeding.

Our results suggest that this methodology is easy and effective to assess the response of hundreds of cassava ovules pollinated with castor bean pollen, facilitating the identification of the development of a multicellular structure in the ES without apparent formation of endosperm. Likewise, cleared-ovules have been successfully implemented for high throughput assessment of *Secale cereale × Zea mays* embryo formation (Ponitka and S´lusarkiewicz-Jarzina, 2004). This methodology proved to be useful to evaluate early stages of embryo and endosperm development after pollination. In some cases, embryos and endosperm were formed, but in others, only embryos or only endosperm was formed. The response was dependent on the rye genotype used. Haploid zygotic embryos that differentiate into fully developed plants had been generated from wide (interspecific/intergeneric) crosses due to a rapid and complete elimination of the genome of the male parent right after pollination (Rajcan et al., 2011). The use of this method for inducing doubled haploids for breeding staple crops is relevant for cassava. Similarly, the methodology optimized by us could be used as a tool for rapid screening and monitoring the response of cassava under different treatments, aiming at optimizing the induction of doubled haploids from unpollinated ovules (*i.e.* gynogenesis, Lentini et al., 2020a), ovules pollinated with irradiated pollen or from interspecific/intergeneric crosses. The advantages of inbreeding for the genetic enhancement of cassava have been reported (Ceballos et al., 2016).

The proposed optimal protocol is illustrated in **Figure 1**. In summary, ovules should be fixed with FAA for at least 8 days followed by dehydration using an ethanol series from 70 to 100% in order to provide the tissues with the proper consistency for sectioning with vibratome. Fixed ovules should then be mounted onto a 7% agarose block using super glue in order to maintain the tissue steady onto the vibratome stage and to increase the control of the samples during the sectioning. Samples should be sectioned under 70% ethanol to avoid curling of the slices, and because fully dehydrated ovules at 100% ethanol are too hard for sectioning. After sectioning with vibratome, slices should be dehydrated further before clearing with methyl salicylate. The proposed protocol is the result of a gradual systematic comparison and modification of various variables in order to provide high-quality images to study cassava ovules. The direct application of previously published protocols for other species was not applicable to cassava. The large size of cassava ovules, the thick integuments, and the effect of the clearing chemical treatment on the tissue consistency were some of the challenges that were addressed in order to optimize a protocol adapted to this species. In contrast to other major crops, cassava is still an orphan staple with limited availability of fundamental information and technology applicable to this crop. This work is a contribution for filling this gap.

## Data Availability Statement

All datasets presented in this study are included in the article/**Supplementary Material**.

## Author Contributions

ZL designed research. ET and MB researched under the supervision of ZL. All authors analyzed data. ZL wrote the paper. All authors contributed to the article and approved the submitted version.

## Funding

This work was funded by the Bill & Melinda Gates Foundation (USA) through the Grant ID No. OPPGD1483. Supporting OPP ID1079312.

## Conflict of Interest

The authors declare that the research was conducted in the absence of any commercial or financial relationships that could be construed as a potential conflict of interest.
