# Supplementary material for: Vibratome Sectioning and Clearing for Easing Studies of Cassava Embryo Formation
Source: Front Plant Sci. 2020 Aug 4;11:1180. doi: 10.3389/fpls.2020.01180 (PMC7417605; doi:10.3389/fpls.2020.01180)
Supplement: Supplementary file 1 [file Table_1.docx]

**Supplement Table 1 | Protocol used for the isolation and culture of cassava ovule pollinated with castor bean (*Ricinus communis*) pollen**

| Ovule Culture | Protocol according to Lentini et al., 2020b |
| --- | --- |
| Disinfection, isolation and culture | Cyathia were surface-sterilized with 70% ethanol for 1 min, followed by 1.5% sodium hypochlorite solution in water, with 3 drops of Tween 20 for 17 min, and rinsed four times with sterile distilled water. In the sterile environment, the cyathia were further dissected under a stereomicroscope (Nikon C-LEDS and cold-light Nikon NI-150). Pistils were cut off from cyathia just above the nectar glands. The stigmas were removed at the level of the style neck. Excised ovaries without nectar glands and without stigmas were cut longitudinally along the carpel walls in three sections containing one ovule each in each loculus. The carpels were cultured with the basal cut end on MS3 solid medium. Cultures were kept at 28-30 ^o^C in the dark. Once the ovules protruded through the carpel walls (usually about 3 to 4 weeks after the start of the culture), ovules were isolated and placed on fresh medium of the same composition with the adaxial side down on the medium. Ovules were subcultured every 4 weeks on fresh medium. |
| MS3 medium | Macro- and micro-elements (mg/L) based on MS medium. Supplemented with: 0.8 mg/L CuSO_4_ x 5H_2_O 0.8; 2.5 mg/L nicotinic acid; 1.2 mg/L pyridoxine; 10 mg/L thiamine; 4 mg/L glicyne; 500 mg/L myo-inositol; 0.2 mg/L biotin; 0.2 mg/L Ca-pantotenate; 0.2 mg/L ascorbic acid; 0.4 mg/L riboflavin; 200 mg/l l-proline; 400 mg/l l-glutamine; 150 mg/l casein hydrolysate; 2.0 mg/l 2,4 d; 2.0 mg/l BAP; 1.0 mg/l ga3; 80 g/l sucrose; and 3 g/l gellam gum. |
